# Mycobacterial Adhesion: From Hydrophobic to Receptor-Ligand Interactions

**DOI:** 10.3390/microorganisms10020454

**Published:** 2022-02-16

**Authors:** Albertus Viljoen, Yves F. Dufrêne, Jérôme Nigou

**Affiliations:** 1 Louvain Institute of Biomolecular Science and Technology, Université Catholique de Louvain, Croix du Sud, 4-5, bte L7.07.07, B-1348 Louvain-la-Neuve, Belgium; yves.dufrene@uclouvain.be; 2 Institut de Pharmacologie et de Biologie Structurale, Université de Toulouse, CNRS, Université Paul Sabatier, 31077 Toulouse, France; jerome.nigou@ipbs.fr

**Keywords:** mycobacterium, host-pathogen interaction, adhesion, bacterial envelope, adhesin, tuberculosis

## Abstract

Adhesion is crucial for the infective lifestyles of bacterial pathogens. Adhesion to non-living surfaces, other microbial cells, and components of the biofilm extracellular matrix are crucial for biofilm formation and integrity, plus adherence to host factors constitutes a first step leading to an infection. Adhesion is, therefore, at the core of pathogens’ ability to contaminate, transmit, establish residency within a host, and cause an infection. Several mycobacterial species cause diseases in humans and animals with diverse clinical manifestations. *Mycobacterium tuberculosis*, which enters through the respiratory tract, first adheres to alveolar macrophages and epithelial cells leading up to transmigration across the alveolar epithelium and containment within granulomas. Later, when dissemination occurs, the bacilli need to adhere to extracellular matrix components to infect extrapulmonary sites. Mycobacteria causing zoonotic infections and emerging nontuberculous mycobacterial pathogens follow divergent routes of infection that probably require adapted adhesion mechanisms. New evidence also points to the occurrence of mycobacterial biofilms during infection, emphasizing a need to better understand the adhesive factors required for their formation. Herein, we review the literature on tuberculous and nontuberculous mycobacterial adhesion to living and non-living surfaces, to themselves, to host cells, and to components of the extracellular matrix.

## 1. Introduction

Adhesion is central to microbial proliferation [1]. It drives formation of biofilms, in which individual microbial cells have greater access to nutrients and protection from environmental stresses [2,3]; it is a prerequisite for the colonization of environmental niches [4], and, for pathogens, adhesion to host tissues and cells constitutes one of the first steps in the establishment of an infection [5,6].

The mechanism of bacterial adhesion to a substrate may involve non-specific macroscopic surface properties, such as surface free energy, charge, or hydrophobicity [7], or, as is often the case in pathogens, specialized surface-localized molecules, called adhesins, may act as effectors of adhesion through interactions with specific host molecules [5]. These adhesins often participate in more elaborate processes than the mere act of binding to a specific substrate or ligand. For example, the adhesin function of type IV pili (T4P) is critical for their role in motility [8]. In staphylococcal septicemia, a set of adhesins employing variations of the “dock, lock, and latch” binding mechanism form exceptionally stable, stress-enhanced bonds, allowing the bacteria to remain adhered to blood vessel walls under high flow rates [9]. The adhesion mechanisms expressed and employed by a particular bacterial species, therefore, appear to be tailored to particular mechanobiological as well as broader physiological needs [10].

Although *Mycobacterium tuberculosis*, the best studied mycobacterial pathogen, expresses a repertoire of adhesins, most of these do not appear to function like the bona fide adhesin virulence factors seen in other pathogenic bacteria that offer a means of adherence under high mechanical stresses. Central to the pathogenicity of mycobacterial pathogens, such as *M. tuberculosis* and *Mycobacterium leprae*, is their ability to invade and proliferate inside host cells, so the emphasis is on targeted host cell entry. However, this may not necessarily be the case for all mycobacteria, in particular an emerging class of nontuberculous mycobacteria (NTM). Here, we review the mechanisms employed by both tuberculous mycobacteria and NTM to adhere to abiotic surfaces, themselves, or their host cells and tissues; their broader implications in biofilm formation and immune evasion; and recent insight into their biomechanical function.

## 2. Where and When Is Adhesion Important in Mycobacterial Pathogenesis?

### 2.1. Tuberculosis and Leprosy

**Figure 1:**
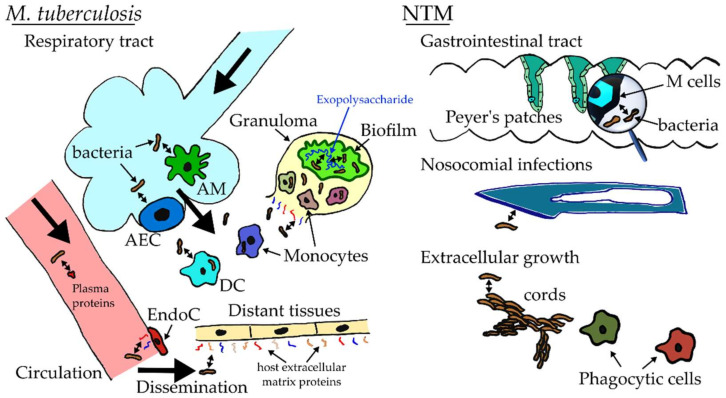
Where and when mycobacterial adhesion occurs during infection. Pulmonary infections involve initial adhesion to alveolar macrophages (AM) or epithelial cells (AEC). After transmigration across the alveolar epithelium bacilli adhere to monocytic cells or dendritic cells (DC). The immune response can contain bacteria in granulomas, where it was very recently demonstrated that *M. tuberculosis* biofilms occur. Upon disintegration of granulomas, bacteria may adhere to extracellular matrix (ECM) components. During hematogenous dissemination, tubercle bacilli may bind plasma fibronectin and, ultimately, to ECM proteins to invade new tissues. Some nontuberculous mycobacteria (NTM) adhere to gut epithelium to cause gastrointestinal infections. NTM that cause nosocomial infections adhere to fomites or surgical equipment. Mycobacteria also self-adhere to form cords, which is a form of immune evasion employed by some NTM. EndoC, endothelial cells.

An overview of mycobacterial adhesion in the clinical context is given in Figure 1. The lifecycle of *M. tuberculosis* starts with the inhalation of small aerosol droplets that were propelled into the air through the cough of a person with active tuberculosis (Tb). Due to their small size, some of these droplets pass the upper respiratory tract and carry tubercle bacilli straight to the alveolar spaces of the lung. These bacteria, thus, get to bypass the competition of the commensal microbial flora and primed microbicidal immunity of the upper respiratory tract [11]. In the alveoli, they are phagocytosed by their preferred host macrophage cells, a process that initiates a complex inflammatory cascade that drives formation of a multicellular structure, called a granuloma, wherein bacilli are contained in a latent infectious phase [12]. Up until here, the adhesion factors used by the tubercle bacilli would seem to mainly consist of surface-exposed lipids and glycoconjugates that bind to a range of receptors expressed on the macrophage surface [13,14]. However, it was discovered that *Mycobacterium bovis* BCG requires the heparin-binding haemagglutinin adhesin (HBHA), which it shares with *M. tuberculosis* and which binds to heparin sulfate-containing receptors on the surface of epithelial cells, for extrapulmonary dissemination in a mouse infection model [15]. This finding suggested that the adhesin may be used by tubercle bacilli to cross the alveolar epithelium, although this is also likely accomplished via diapedesis in the transmigration of infected alveolar macrophages [16]. In certain individuals, especially young children and those with a suppressed immune system, intense intracellular bacillary multiplication during the initial phase of infection or lesion of granulomas, upon reactivation of a long latent infection, leads to lymphatic or hematogenous metastasis [17]. During this metastatic spread to distant sites in the body (nervous system, bones, genitourinary system, skin), the tubercle bacilli probably require adherence to host extracellular matrix components to overcome colonization-hindering mechanical shear forces [18].

Although *M. leprae* is an obligate intracellular pathogen affecting mostly peripheral zones of the body, one of its primary routes of infection is the nose [19], and its ortholog of HBHA has also been implicated in its ability to attach to airway epithelial cells [20]. In addition, the ability of this mycobacterial pathogen to invade the peripheral nervous system has been attributed, at least in part, to the interaction between a yet unidentified adhesin and the G domain of the laminin-α2 chain (LN-α2G) [21]. This would facilitate attachment of *M. leprae* to Schwann cells via a ternary interaction, where LN-α2G forms a bridge between the bacterial adhesin and β4 integrin on the Schwann cell.

### 2.2. Zoonotic and Opportunistic Infections and Emerging Mycobacterial Pathogens

*M. tuberculosis* and *M. leprae* have historically been the most important mycobacterial pathogens, and their intracellular lifestyle may be seen as a paradigm of mycobacterial pathogenicity. Yet, several less important mycobacteria, causing mainly animal disease, and emerging pathogenic nontuberculous mycobacteria (NTM) appear to follow infective lifestyles that deviate from that of tuberculous and leprous bacilli, and, wherein, the mechanisms of adhesion are probably different [22,23]. *Mycobacterium bovis*, the causative agent of bovine tuberculosis, which can also cause zoonoses, can, in addition to an infection resembling pulmonary tuberculosis, cause gastrointestinal infections in humans after consumption of contaminated unpasteurized dairy products [24]. In the NTM *Mycobacterium avium* subsp. *paratuberculosis*, which causes gastrointestinal infections in ruminants known as Johne’s disease and which has been theorized to be associated with human inflammatory bowel diseases such as Crohn’s disease [25], a fibronectin-binding adhesin has been implicated in its ability to bind and penetrate intestinal mucosal epithelium [26,27]. This was found to involve high-density integrin-displaying and, therefore, fibronectin-binding M cells [28]. The role of adhesion in NTM physiopathology, often associated with environmental sources of contamination and distinct extracellular phases, is underexplored, and should be given more attention. These include the skin pathogens *Mycobacterium ulcerans* and *Mycobacterium marinum*, although for the former the role played by adhesins during cutaneous infection is disputed [29], as well as for a number of NTM that are associated with surgical procedure–related infections, including *Mycobacterium chelonae*, *Mycobacterium fortuitum*, and members of the *Mycobacterium abscessus* complex (MAC) [30]. The nosocomial nature of these infections involves adhesion in the contamination of fomites, including surgical equipment, and subsequent transmission onto host tissues [31,32,33]. It is worth noting that MAC also causes human transmissible pulmonary infections for which cystic fibrosis sufferers show a heightened vulnerability [22,34,35].

### 2.3. Adhesive Interactions in Mycobacterial Biofilms

The capacity of various environmental mycobacteria (including species that can cause human infections) to form robust biofilms in sources such as domestic water distributions systems [36,37,38] and medical equipment [39,40] has been known for several decades. Evidence also exists of the occurrence of mycobacterial biofilms in vivo during the course of infection for some NTM species [41,42,43,44,45]. However, the direct involvement of *M. tuberculosis* biofilms during the course of tuberculosis was largely considered to be non-existent until very recently. Reports of such biofilms in animal models of infection, including nonhuman primates, as well as in histological lung sections from human tuberculosis patients, implicate the formation of these structures in pathogenesis [46,47]. These biofilms were further demonstrated to contribute in the resistance of resident tubercle bacilli to both antitubercular treatments and the human immune response [47]. Understanding how these mycobacterial biofilms are formed in vivo may pave the way to the discovery of new and better therapeutic strategies to treat mycobacterial infections. We point out that adhesion plays at least three key roles in biofilm formation: In the first instance, adhesion of planktonic bacteria to a substratum serves as the point of nucleation and, thus, as the very first step in biofilm development. Secondly, intercellular adhesion is very likely required during early growth of the biofilm. Third, the biofilm extracellular matrix, which holds individual cells together and which constitutes the major biomass component in mature biofilms, relies on adhesive interactions to provide mechanical stability to the mature biofilm [48].

**Figure 2:**
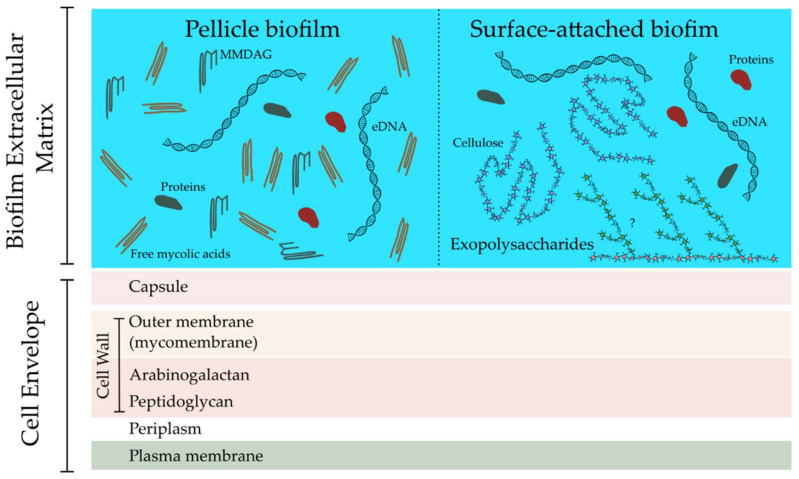
The composition of the biofilm extracellular matrix is different in pellicle biofilms and surface-attached biofilms. While the former is rich in free mycolic acids and monomeromycolyl diacylglycerol (MMDAG), the latter is lipid-poor and contains large amounts of exopolysaccharides. Both types contain extracellular DNA (eDNA) and proteins.

In routine axenic broth cultures, mycobacteria generally grow as pellicle biofilms, types of biofilms that form at the air-liquid interface and that are distinct from classical biofilms, which are surface-attached. Most of what is known for *M. tuberculosis* biofilms is based on data for the pellicular form (Figure 2). Principal components of the mycobacterial pellicle biofilm extracellular matrix are extracellular DNA [49,50,51] and free mycolic acids [52,53]. In addition, a range of lipids including short chain mycolic acids, monomeromycolyl diacylglycerol, mycolate ester wax, glycopeptidolipids (GPLs), phthiocerol dimycoserosates (PDIM), phenolic glycolipids (PGL), and keto mycolic acids have been directly implicated in the ability of mycobacteria to form pellicle biofilms [53,54,55,56,57,58]. Although some of these lipids were also reported to be constituents of the biofilm extracellular matrix, it has been proposed that their major participation in biofilm formation is more likely to be in direct interbacterial adhesion and involves their hydrophobicity (at least in the case of the less amphiphilic ones) [59]. While exopolysaccharides are abundant in the extracellular matrices of many microbes [60,61,62,63,64], evidence of these complex carbohydrates in mycobacterial biofilms have been lacking. Very recently, a rapidly inducible surface-attached in vitro biofilm model was devised for *M. tuberculosis* [47,65]. Interestingly, it was discovered that the principal components of these biofilms were exopolysaccharides (Figure 2), among which cellulose was identified [65]. The presence of cellulose in the biofilm extracellular matrix was also reported for *M. avium*, *Mycobacterium fortuitum*, and *M. smegmatis* [65,66]. The detection of mannose and galactose in the composition of *M. ulcerans* [43] and *M. smegmatis* [67] biofilm extracellular matrices, respectively, in addition to glucose, may hint towards the presence of yet unidentified exopolysaccharides. Importantly, cellulose was also detected in mycobacterial biofilms occurring during infection [65], pointing to a role for biofilm extracellular matrix exopolysaccharides in pathophysiology. It needs to be addressed how the exopolysaccharide components of mycobacterial biofilms contribute to structural integrity of the latter. Another important question to be addressed is what are the mycobacterial surface components that bind to these exopolysaccharides. A number of recent reports of bacterial lectins binding biofilm extracellular matrix exopolysaccharides [68,69,70,71,72,73,74] intimate that such lectins may also be present in mycobacteria.

## 3. Non-Specific Adhesion: The Hydrophobic Mycobacterial Surface

Recent reports have highlighted the role of surface hydrophobicity in mycobacterial pathogenicity, more specifically how *M. tuberculosis*’s evolution from a non-pathogenic environmental ancestor to an obligate and highly successful human pathogen positively correlates with surface hydrophobicity [75,76,77,78]. According to the model given by the authors of these studies, the evolutionary loss of relatively hydrophilic lipids such as lipooligosaccharides (LOSs) and acquisition of highly hydrophobic lipids such as PDIMs, pentaacyl trehaloses (PATs), and sulfoglycolipids (SGLs) in modern *M. tuberculosis* strains improved their aerosolization and, hence, their transmission [75,77].

A clue for another way in which a very hydrophobic cell surface may contribute to mycobacterial virulence is brought by the rapidly growing NTM *M. abscessus*, for which concern is mounting over the human transmissible infections that it is causing, which are particularly difficult to manage in cystic fibrosis sufferers [34,35,79,80]. *M. abscessus* normally produces a class of polar lipids, called GPLs, that accounts for a comparatively hydrophilic surface. In mutants lacking a component of the GPL biosynthetic and transport machinery, the cell surface is as hydrophobic as that of *M. tuberculosis*, most likely because of the surface exposure of hydrophobic lipids such as trehalose mycolates and trehalose polyphleates [81,82,83]. These lipids have been implicated in the ability of mycobacteria to form cords, which is strongly associated with mycobacterial virulence [84,85,86]. For *M. abscessus*, the transition from being GPL^+^ to being GPL^−^ has been reported to occur during the course of infection and is associated with more severe disease [87,88]. Studies using zebrafish embryos as an infection model have attributed the increased virulence of GPL^−^ *M. abscessus* strains to their ability to form cords and the inability of macrophages to engulf these cords [89,90]. A biophysical explanation for cording lies in the dehydrating capacity of the hydrophobic cell surfaces that removes the vicinal water film [91]. This results in a greatly increased density of direct contacts between closely apposed cell surfaces and, hence, strong interbacterial adhesion. The adhesive effect of hydrophobic surfaces even counts for contacts with relatively hydrophilic surfaces and the role of bacterial surface hydrophobicity in adhesion to host tissues as well as in phagocytic ingestion has been known for a long time [92,93]. We point out, based on our own laboratory experience, that even abundant mycobacteria-producing polar lipids such as LOSs and GPLs are, nevertheless, considerably more hydrophobic than other Gram-positive and Negative species, such as *Staphylococcus aureus*, *Pseudomonas aeruginosa*, and *Escherichia coli*. Even non-mycobacterial mycolic-acid-containing species, such as *Corynebacterium* and *Gordona*, exhibit considerable surface hydrophobicity [94]; in all fairness, hydrophobic mycobacteria, such as *M. tuberculosis* and GPL^−^ *M. abscessus*, are to be considered extremely hydrophobic.

Hydrophobicity, therefore, plays a critical role in inter-mycobacterial adhesion, which in turn is a requirement for cord formation, a form of immune evasion in some pathogenic species. The hydrophobic cell envelope also probably contributes to the contacts that are instated between mycobacterium and macrophage during phagocytic engulfment by likely facilitating specific interactions between cell receptors and their cognate bacterial-surface ligands. In the next section we will dive deeper into the mycobacterial factors that underlie adhesion to specific host factors (also summarized in Table 1).

## 4. Controlling Host Cell Adhesion: Molecules That Bind to Cells and Tissues

### 4.1. Interactions with Immune Cell Receptors: Ligands and Surface Distribution

The recognition of mycobacterial lipids and glycoconjugates by immune cell receptors has been the subject of several recent reviews and book chapters [13,14,112,113,114,115,116,117], so to avoid being repetitive we will only provide a brief summary of some key players in these interactions and discuss salient points relating to pathogen-cell adhesion.

A great variety of mycobacterial surface components (some of which are also plasma membrane localized) interact with receptors expressed on host cells. These include mycolic-acid-containing glycolipids such as trehalose dimycolate (TDM), glucose monomycolate (GMM), and glycerol monomycolate (GroMM); the lipoglycans phosphatidyl-*myo*-inositol mannosides (PIMs), lipomannan (LM) and lipoarabinomannan (LAM); and di- and tri-acyl-trehalose (DAT, TAT), SGLs, PGLs, GPLs [13,14], capsular glucans (most abundant in *M. tuberculosis*), and lipoproteins (most abundant in some NTM) [118,119]. The major pattern recognition receptors (PRRs) that have been identified so far to recognize these mycobacterial microbial-associated molecular patterns (MAMPs) are toll-like receptors (for example, TLR2 binds lipoglycans and lipoproteins), C-type lectin receptors (for example, Mincle binds TDM, GMM, GroMM, DAT, and TAT, while Dectin-2, Mannose receptor, and DC-SIGN bind lipoglycans), and scavenger receptors (for example, MARCO binds TDM). Binding of these MAMPs to their PRRs plays a central role in both the host’s immune response to infection and the ability of mycobacterial pathogens to evade and modulate immunity. In *M. tuberculosis*, PDIMs mask its TLR2-binding MAMPs, hence avoiding recruitment of microbicidal macrophages, while it exposes PGL (in some isolates of the hypervirulent W-Beijing family [120]). This stimulates chemokine-dependent recruitment of permissive macrophages that it binds to and infects in the alveoli [11,121]. Similarly, in the NTM *M. abscessus* GPLs mask underlying MAMPs preventing their proinflammatory interaction with TLR2 [122,123], although in *M. avium* certain serovar-specific GPLs themselves are proinflammatory [124]. In addition to masking TLR2 agonists, it was recently discovered that *M. tuberculosis* negatively modulates TLR2-dependent NF-κB activation and succeeding cytokine or costimulatory molecule production using SGLs as competitive TLR2 antagonists [125]. Both PDIMs and SGLs are considered virulence factors of *M. tuberculosis*, and together they impede TLR2 functioning such that non-permissive phagocytic cells are not recruited towards cites of infection.

In the context of adhesion, a distinction needs to be made between PRR-MAMP interactions that likely support an adhesive contact between bacterium and host cell and those that are less likely to do so. For example, interaction of the TLR2/TLR1 or TLR2/TLR6 heterodimeric complexes with their MAMP ligands involves direct recognition of the MAMP lipidic chains, which in the case of TLR1 and TLR2 dock within channels found inside the PRRs (reviewed here [116,126]). This implies that these MAMPs need to be extracted from the outer cell envelope layers to bind these PRRs and hence it is hard to conceive how they would remain anchored to the bacterial cell during the interaction. Indeed, in the case of lipopolysaccharide (LPS), an LPS-binding protein (LBP) and the scavenger receptor CD14 play consecutive roles in extracting LPS from the Gram-negative bacterial outer membrane and transferring it to the TLR4/MD-2 complex [127]. CD14, in conjunction with CD36, carries out a similar function in the transfer of diacylglycerol ligands onto TLR2/TLR6 or TLR2/TLR1 heterodimers [128]. This role has also been attributed to CD14 in the presentation of mycobacterial lipoglycans to TLR2/TLR1 [129,130]. We argue that such interactions, while important for PRR-signalling, cytokine responses, and macrophage recruitment, are probably not involved in establishing a direct contact between pathogen and phagocytic cell and ultimately do not play a mechanical role during engulfment. On the other hand, C-type lectin PRRs such as Mannose receptor, Dectin-2, and DC-SIGN bind to head groups of their associated MAMPs and do not require their extraction. This means that the latter molecules can remain anchored to the cell envelope while interacting with their cognate PRRs and can thus offer a means of adherence between pathogen and host cell during phagocytic engulfment. Indeed, binding data between whole mycobacteria and these receptors (soluble-recombinant, membrane-expressed in cell lines, or physiological cells) have been obtained (see for example [95,96,131,132,133,134,135]).

**Figure 3:**
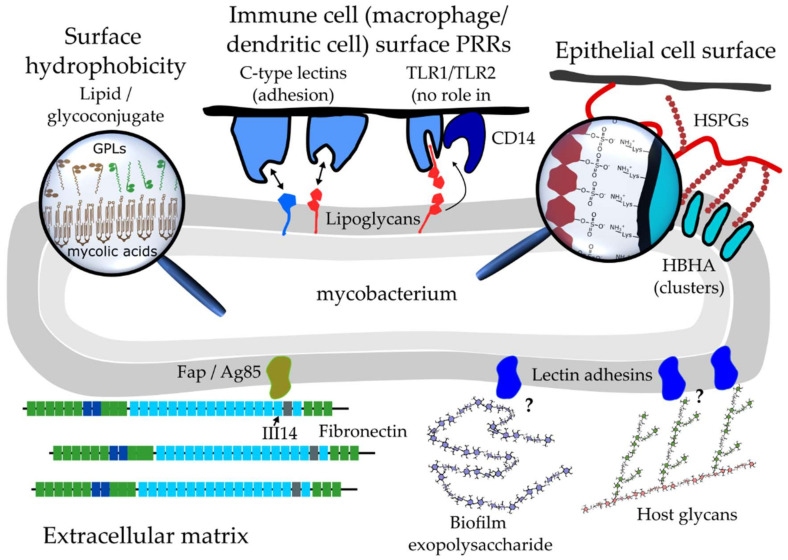
How mycobacteria control adhesion. Sequestration of lipids into nanodomains on the cell surface, as observed for glycopeptidolipids (GPLs) in *Mycobacterium abscessus* [81,137], may control hydrophobic interactions with foreign surfaces. In the same line, the surface exposure of specific microbial-associated molecular patterns (MAMPs) control interactions with pattern recognition receptors (PRRs). Here a distinction needs to be made between MAMP-PRR interactions that facilitate adhesion and those that play other roles. For example, TLR1/TLR2 interact with the hydrophobic acyl chains of their MAMP ligands, requiring their extraction out of the mycobacterial surface layers by accessory molecules such as CD14. Hence, the lack of an anchorage point on the bacterial surface disqualifies these interactions from playing a direct part in adhesion. On the other hand, PRR’s such as certain C-type lectins, including the mannose receptor, DC-SIGN and Dectin-2 bind the saccharide head groups of mycobacterial MAMPs whose hydrophobic acyl chains remain anchored within outer envelope layers. Such interactions play a direct role in mycobacterium-host cell binding and subsequent internalization of the bacteria. Specialized surface proteins called adhesins bind to specific host factors. Clustering of adhesins, such as heparin-binding haemagglutinin adhesin (HBHA), increases avidity of their interactions with heparin sulfate proteoglycans (HSPG), enhancing adhesion to epithelial cells. Adhesins, such as the fibronectin attachment protein (Fap) and members from the antigen 85 complex, drive adhesion of extracellular matrix components. We hypothesize that yet-unknown-carbohydrate-binding (lectin) adhesins bind to exopolysaccharide components of the biofilm extracellular matrix and/or to host glycans.

Regarding the PRR-PAMP interactions that likely do underlie direct contacts between pathogen and host cell as well as likely play a mechanical role during phagocytic engulfment, variations in the structures of mycobacterial MAMPs are important and probably contribute to inter-mycobacterial differences in adhesion to host cells. A prime example is LAM, for which the mannose-capped form (ManLAM) present in pathogenic tuberculous mycobacteria is recognised by the C-type lectins Mannose Receptor [97], Dectin-2 [95,96] and DC-SIGN [98]. Inositol phosphate- (*M. smegmatis* and *M. fortuitum*) and arabinose-terminated LAM (*M. chelonae*) from NTM are not ligands of these receptors [95,96,98,99,131,136]. The number of specific mycobacterial MAMPs recognized by these C-type lectin can vary considerably. It was recently shown using a variety of purified and synthetic mannoconjugates, as well as *M. tuberculosis* isogenic mutant strains, that ManLAM is the sole mycobacterial ligand of Dectin-2. ManLAM recognition requires dimannoside caps and involves multivalent interactions in line with earlier crystallographic observations indicating two monosaccharide binding sites in Dectin-2′s carbohydrate binding domain that allow interaction with dimannosides [96]. In contrast, DC-SIGN binds several ligands in addition to ManLAM, including capsular α-glucan [99], which is abundant in tuberculous mycobacteria but not in NTM, and mannosylated (lipo)glycoproteins from *M. tuberculosis* [131]. Despite a selective recognition of *M. tuberculosis* complex strains, DC-SIGN, surprisingly, also binds LM [131] and hexamannosylated PIM [136], both of which are ubiquitously present in mycobacteria. In addition, certain NTM expressing ManLAM poorly bound to DC-SIGN-expressing HeLa cells in comparison to *M. tuberculosis* complex species, while ManLAM purified from these NTM efficiently inhibited *M. tuberculosis* binding to cellular DC-SIGN [131]. These observations indicate that localization of the various DC-SIGN ligands on the surfaces of mycobacteria may be a determinant in their interaction with DC-SIGN expressing cells, including dendritic cells. It raises the question whether the surface distribution of mycobacterial lipids and glycoconjugates play a role in their recognition by host cells. Interestingly, recent high-resolution atomic force microscopy (AFM) studies revealed that the presence of GPLs account for defined hydrophilic nanodomains on the surfaces of *M. abscessus* cells [81,137]. It is, thus, possible that lateral partitioning of surface components into nanodomains is a factor in their recognition by host cell receptors (Figure 3).

### 4.2. Multifunctional Mycobacterial Adhesins

We alluded earlier to the role that HBHA plays in the early stages of infection, where adhesion of *M. tuberculosis* to alveolar epithelial cells is mediated by binding of the adhesin to heparan sulfate proteoglycans (HSPGs) [15,100,101,102]. After export by a leader peptide-independent mechanism, it remains unclear whether or how HBHA is anchored to the mycobacterial cell envelope, although it may involve binding of an N-terminal sequence to the transmembrane proteins Rv0613c or MmpL14 [138]. Binding to HSPGs occurs via electrostatic intermolecular bridges between C-terminal lysine residues in HBHA and the sulfate groups of heparin sulfate [139]. AFM molecular recognition experiments revealed that HBHA clustered in nanodomains on top of *M. bovis* BCG cells in contrast to a homogenous distribution of the HSPG receptors on lung epithelial cells [140]. Clustering of HBHA on the mycobacterial cells may serve to strengthen adhesion to epithelial cells by promoting the formation of multiple parallel bonds (Figure 3), a common behaviour for other bacterial and fungal adhesins [141]. Another adhesive role was proposed for HBHA in mycobacterial agglutination involving homophilic interactions between two N-terminal coiled coils [142]. As is the case for most mycobacterial adhesins identified so far, HBHA plays additional roles to that of an adhesin: it has a cytosolic function in the formation of intracytoplasmic lipid inclusions [143]; it also has been implicated in the reorganization of actin filaments within epithelial cells [144] involving direct binding to actin [145]; and it induces apoptosis in macrophages involving the endoplasmic reticulum [146] and mitochondria [147].

Several mycobacterial proteins have been identified that bind host extracellular matrix proteins, such as fibronectin, collagen, and laminin. Of these, the best studied are two classes of fibronectin-binding adhesins. The fibronectin attachment protein (FAP, also known as the alanine and proline-rich secreted glycoprotein Apa or antigen MPT-32), binds fibronectin using a 12-amino acid minimal binding sequence that was originally mapped in the *M. avium* ortholog (G^269^NRQRWFVVWLG^280^, the underlined sequence is essential for fibronectin binding), which is widely conserved among mycobacterial species [103,104,105]. Although the specific fibronectin sequence that FAP binds to is unknown, inhibition of binding by heparin indicates that it must be located within one of its C-terminal heparin-binding domains (III_12–14_) [105]. FAP has been implicated in adhesion to host tissues in multiple mycobacterial species: the *M. leprae* ortholog of FAP was implicated in its capacity to invade both epithelial cells and Schwann cells [104], that of *M. bovis* BCG was found to be necessary for its attachment to the bladder wall and for *M. bovis* BCG-induced antitumor activity [148,149,150], *M. avium*’s ortholog was implicated in bacterial adhesion to fibrous human respiratory mucosa [151], FAP of *M. avium* subsp. *paratuberculosis* mediated adhesion to intestinal epithelial and M cells [26,27,28,152], and the *M. tuberculosis* ortholog was implicated along with antigen 85B (Ag85B) in bacterial adhesion to human respiratory mucosa [153], although *M. tuberculosis* respiratory mucosa infection was found to be independent of fibronectin attachment [154]. Concerning additional roles, it was found that the *M. avium* subsp. *paratuberculosis* FAP ortholog activates dendritic cells [155], probably using a fibronectin-independent mechanism involving binding to DC-SIGN, since the *M. tuberculosis* ortholog is a ligand of this receptor [131].

The second family of well-characterised mycobacterial fibronectin-binding proteins is the antigen 85 (Ag85) complex. In addition to their fibronectin-binding activity, these proteins play a crucial role in the synthesis of the mycomembrane through their essential mycolyltransferase activity [156,157], except for a paralog originally identified in *M. tuberculosis*, which lacks this activity [157,158]. Due to the essential role that members of the Ag85 complex play in cell envelope synthesis, they are universally conserved among mycobacteria and essential for survival, and, therefore, efforts have been made to exploit them for the development of new antimicrobials specifically targeting mycobacteria [159,160,161,162,163,164]. The fibronectin-binding sequences that are highly conserved among mycobacterial species were identified in *M. tuberculosis* and *M. avium* subsp. *paratuberculosis* Ag85A, Ag85B, and Ag85C (F^101^EWYNQSGISV^111^, F^98^EWYYQSGLSV^108^, and F^102^EEFYQSGLSV^112^, respectively for the latter species) along with the sequence that they bind in fibronectin, which mapped to repeat domain module III_14_ (T^14^PNSLLVSWQPPR^26^) [106,107]. Although FAP may also bind near this location, it is unlikely that the specific binding sequence is shared based on the apolarity and positive charges of residues in FAP’s minimal fibronectin-binding sequence in contrast with the abundance of polar and negatively charged residues in that of Ag85. Although early studies demonstrated a high specificity towards binding fibronectin among extracellular matrix proteins, it was found that *M. tuberculosis* Ag85 also binds human tropoelastin, by means of a unique binding mechanism [165].

Several additional mycobacterial adhesins have been identified that bind host extracellular matrix proteins [166,167,168,169,170]. To name a few briefly, these include Rv1759c (a PE_PGRS that binds fibronectin) [171], malate synthase (a glyoxalate shunt enzyme that is secreted by an unknown mechanism and that binds laminin as well as fibronectin) [172], and GAPDH (a glycolytic enzyme that binds plasminogen and plasmin with relatively high affinity hence offering a means to degrade extracellular matrix components) [173].

### 4.3. Appendages and Lectins

The *M. tuberculosis* genome contains genes for two types of pili. The first type, for which only a single gene (encoding the pilin subunit) has been identified, is known as the *M. tuberculosis* pilus (Mtp) and presents morphological and biochemical characteristics reminiscent of curli amyloids [111,174]. The second type is encoded by a reduced set of tight adherence (Tad) pilus genes (5 out of the 14 *Aggregatibacter actinomycetemcomitans Tad pilus* genes, in which the genetics of this class of type IV pili was first characterized) [175]. Mtp have been observed in a small number of studies by method of transmission electron microscopy or AFM [111,175,176,177,178]. Mtp binds laminin [111] and has been implicated in the ability of *M. tuberculosis* to invade epithelial and macrophage cell lines in culture [179,180], but inactivation of *mtp* in two *M. tuberculosis* strains resulted in no change in the outcome of infection in C3HeB/FeJ mice that form necrotic, hypoxic lesions in which adherence to extracellular matrix proteins could play a role in mycobacterial colonization [175]. Nevertheless, recent studies have implicated Mtp deficiency in metabolic alterations in *M. tuberculosis* [181], macrophages [182], and epithelial cells [183]. Unlike Mtp, the mycobacterial Tad pilus has only been observed microscopically in one study and in its heterologously-expressed and purified crystalline form [174], but like for *mtp* inactivation, deletion of *M. tuberculosis tad* genes had no effect on virulence [175]. A surprising aspect of both Mtp and *Rv3654c* (encoding the Tad pilin) is the apparent absence of functional orthologs among NTM, which for Mtp along with its antigenicity has led to it being explored as a biomarker in a diagnostic test for *M. tuberculosis* complex species [184].

Another set of mycobacterial surface proteins that may play a role in cellular adhesion via their carbohydrate-binding activities are lectins. Recent functional and structural studies have highlighted the plasticity and adaptivity of glycan-mediated host–pathogen interactions [185]. The host glycome encompasses an enormous complexity in intracellular and extracellular monosaccharides, oligosaccharides, and polysaccharides and their glycoconjugate derivatives (glycolipids and glycoproteins). It plays a key role in cell and tissue recognition and physiology, but also forms a frequent interaction site for colonization by bacterial pathogens such as *Pseudomonas aeruginosa* [186], *Escherichia coli* [187], *Helicobacter pylori* [188], and *Salmonella enterica* [189], to name a few. Although they were subject of a recent review [190], very little remains known about mycobacterial lectin genetics, biochemistry, or involvement in pathogenesis. The *M. tuberculosis* genome encodes only 11 putative lectins [191] and of these only 2 have been characterised biochemically, one being HBHA (that we discussed earlier), whose binding to sulfated glycoconjugates depends on electrostatic interactions between charged lysine residues and the sulfate groups, rather than a characteristic carbohydrate recognition domain [192], and the secreted 13 kDa ricin-like lectin (sMTL-13). The latter shows antigenic activity in Tb patients [110]. Early studies identified a 14 kDa lectin apparently conserved between *M. smegmatis*, *M. tuberculosis*, *M. leprae*, *M. kansasii*, and *M. avium*, with hemagglutinating activity and a role in adhesion to mouse peritoneal macrophages, both activities that could be inhibited by mannan [193,194]. The polypeptide sequence of this lectin and the gene encoding it have not been identified. Regarding NTM, crystal structures of the β-prism II fold lectin domain of *M. smegmatis* MSMEG_3662 in apo form as well as in complex with mannose and methyl-α-mannose were solved [108,109]. While this lectin has no apparent orthologs in *M. tuberculosis*, an ortholog (with 87% identity) is present in *M. abscessus*, thus making future investigations of its role in pathogen-host interaction worthwhile.

## 5. Mycobacterial Adhesion under Mechanical Stress

**Figure 4:**
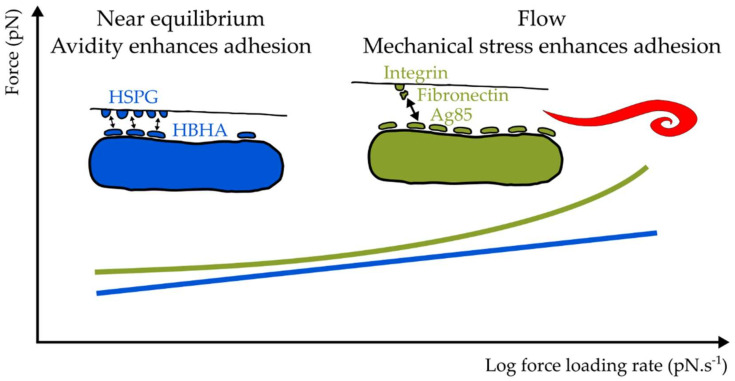
Mechanical stress enhances Antigen 85 (Ag85) binding to fibronectin, a possible mechanism to enhance adhesion in dynamic environments such as the circulatory system or in the gastrointestinal tract. On the other hand, HBHA binding HSPG receptors on alveolar epithelial cells follows conventional forced unbinding kinetics. In this case, adhesion is enhanced through HBHA clustering allowing multiple parallel interactions with HSPG.

The search string “(((((((mycobacterium) OR (mycobacteria)) OR (mycobacterial)) OR (bacterium)) OR (bacterial)) OR (bacteria)) AND (adhesion)) AND (shear)” delivers in excess of 795 hits on https://pubmed.ncbi.nlm.nih.gov/, (accessed on 20 December 2021) and of these only two concern a mycobacterial species [195,196]. Is this because of a lack of interest, or simply because mechanical shear stress is not a significant obstacle encountered by these pathogens during infection? A common feature in practically all known mycobacterial surface molecules that play a direct role in adhesion is that they are non-covalently (and apparently often loosely) associated with the mycobacterial surface. In Gram-positive taxa, including *Bacillus*, *Clostridia*, *Enterococcus*, *Lactobacillaceae*, *Listeria*, *Staphylococcus*, *Streptococcaceae*, and even in *Actinobacteria*, such as the genera *Corynebacterium* and *Streptomyces*, a sortase enzyme covalently ligates secreted proteins baring a conserved recognition sequence, including adhesins, to the pentaglycine cross bridges in peptidoglycan, firmly anchoring these proteins to the cell. Based on a pblast (https://www.genome.jp/tools/blast/, accessed on 20 December 2021) of *S. aureus* SrtA against all mycobacterial genomes in the KEGG database, these genera do not seem to possess any sortase genes. To our knowledge, no sortase activity has ever been reported for any mycobacterial species. Peptidoglycan-anchored adhesin-host factor complexes can withstand considerable shear forces, with extreme examples uncovered recently in staphylococci that resist tensile forces under which covalent bonds can rupture (in excess of 2000 pN for a single molecular complex under physiologically relevant rates of force application) [197,198,199,200]. The small number of force spectroscopy studies that have been done on mycobacterial adhesins interacting with their ligands revealed much weaker mechanical stabilities. For example, single molecular complexes of HBHA and heparin sulfate or actin, when pulled apart under velocities that resemble blood flow rates, ruptured at approximately 50 pN and 60 pN, respectively [139,145]. Similarly, single molecular complexes of *M. bovis* BCG adhesins and fibronectin ruptured under tensile forces of ~50 pN [201]. The relative tensile weakness of these interactions may be a result of the non-covalent anchorage of the adhesins to the cell envelope, which has resulted in different evolutionary mechanisms to maximize adhesion. Fluid shear was indeed found to enhance *M. tuberculosis* adhesion on fibronectin or surfactant protein A-coated surfaces [195], and a recent force spectroscopy study found that the mechanical stability of the complex formed by *M. abscessus* Ag85 and fibronectin correlates unconventionally with the rate at which force is applied (loading rate) (Figure 4) [196]. As a consequence, unusually strong bonds (~150 pN) were observed under high loading rates. Interestingly, this contrasts HBHA, whose interaction with heparin sulfate follows conventional forced unbinding kinetics (force scales linearly with the logarithm of force loading rate) [139]. These observations do indicate that at least some mycobacterial adhesins are adapted to bind their ligands under non-equilibrium dynamic shear conditions.

## 6. Conclusions and Future Perspectives

The ability of mycobacteria to adhere to surfaces in the clinical environment, to host cells or to extracellular matrix components lining mucosal epithelia is an important issue to address in controlling infections with these pathogens. Despite this, very little effort has been made to develop specific inhibitors of mycobacterial adhesion. In this regard, peptide sequences that mimic the binding sites in adhesins or their ligands may hold promise. For example, synthetic peptides of the minimal binding sequences in the fibronectin-Ag85 interaction could efficiently block adhesion of *M. avium* subsp. *paratuberculosis* and *M. abscessus* cells to fibronectin surfaces [106,196]. More recently, a short peptide sequence from the novel *M. tuberculosis* hyaluronic acid-binding adhesin Rv3194c, could inhibit *M. tuberculosis* binding to an epithelial cell line [202]. Another promising avenue that may be explored is that of synthetic ligands of PRRs involved in phagocytic engulfment of mycobacteria as inhibitors of mycobacterial adhesion [96,203,204,205]. However, although these strategies targeting specific interactions should be evaluated for their antiadhesive potential in mycobacterial infection models, a problem that might be encountered is that of redundancy brought by the large diversity of mycobacterial molecules that bind host factors. In that regard, we point out that most (if not all) mycobacterial surface molecules that bind to host factors appear to be loosely attached to the mycobacterial envelope. Their presence on the surface of mycobacteria is likely strongly dependent on the integrity of the mycomembrane. We speculate that compounds interfering in the assembly of the mycomembrane would strongly impact on the interactions between surface molecules and their cognate receptors (in the case of MAMPs) or ligands (in the case of adhesins). We recently reported that inhibition of mycolic acid transport in *M. abscessus* led to rapid and dramatic decreases in surface hydrophobicity, suggesting considerable alterations of surface chemical properties caused by the treatment.

Although a variety of mycobacterial adhesive molecules, including MAMPs and adhesins, have been identified and their interactions with host factors characterised, several questions have not been addressed. These include: (i) How are these molecules attached to the mycobacterial surface and how strong are these attachments. (ii) The distribution of lipids and glycoconjugates in the different layers of the mycobacterial cell envelope has been extensively studied (see, for example [206]). However, very little remains known on the lateral distribution of these molecules on the surface where they may participate in adhesive interactions. How does their lateral distribution change as a function of growth or environmental fluctuations or stressors? (iii) What are the major players in adhesion in *M. tuberculosis* (a question that remains under investigated), as well as in emerging NTM pathogens such as MAC species? (iv) How are surface adhesive properties regulated and in response to which stimuli? (v) What are the out-of-equilibrium (mechanically stressed) binding dynamics of interactions between mycobacterial adhesive molecules and their cognate binding partners? Future studies should be devoted to addressing these outstanding questions. Such studies will largely benefit from recent advances in super-resolution microscopy and AFM force spectroscopy techniques, whose combined capabilities have led to massive advancements in our understanding of pathogen (bacterial and viral) adhesion in recent years [207,208,209].

## Figures and Tables

**Table 1:** Major known mycobacterial adhesive molecules and their host factor targets.

Molecule Class (Examples)	Host Factor(s)	Key References
Lipids/glycoconjugates:		
mannose-capped lipoarabinomannan (ManLAM)	Pattern recognition receptors/C-type lectins (Mannose receptor, DC-SIGN, Dectin-2)	[95,96,97,98]
α-glucan	DC-SIGN	[99]
Adhesins:		
heparin-binding haemagglutinin adhesin (HBHA)	heparan sulfate	[15,100,101,102]
fibronectin attachment protein (Fap)	fibronectin	[103,104,105]
antigen 85 (Ag85) complex	fibronectin	[106,107]
Lectin adhesins:		
β-prism II fold lectin	Unknown	[108,109]
13 kDa ricin-like lectin (sMTL-13)	Unknown	[110]
Appendages:		
*M. tuberculosis* pilus (Mtp)	lamanin	[111]

## Data Availability

Not applicable.
